# Prevalence and prognostic value of activated protein C resistance and anti–protein C antibodies in patients with aPLs: an APS ACTION Registry study

**DOI:** 10.1093/rheumatology/keag304

**Published:** 2026-06-13

**Authors:** Ibrahim Tohidi-Esfahani, Veronica Venturelli, Maria Tektonidou, Vittorio Pengo, Diana Parades-Ruiz, D Ware Branch, Maria Gerosa, Cecilia Nalli, Esther Rodriguez Almaraz, Michelle Petri, Ricard Cervera, Olga Amengual, Danieli de Andrade, Rohan Willis, Maria Laura Bertolaccini, Doruk Erkan, Hannah Cohen, Maria Efthymiou

**Affiliations:** Department of Haematology, University College London, London, UK; Department of Haematology, Concord Repatriation General Hospital, Sydney, Australia; Platelet and Thrombosis Research Laboratory, ANZAC Research Institute, University of Sydney, Sydney, Australia; Department of Haematology, University College London, London, UK; Centre for Rheumatology, Department of Medicine, University College London, London, United Kingdom; Rheumatology Unit, Department of Medical Sciences, Università degli Studi di Ferrara, Azienda Ospedaliero-Universitaria S. Anna, Cona, Italy; Rheumatology Unit, First Department of Propaedeutic and Internal Medicine, School of Medicine, National and Kapodistrian University of Athens, Athens, Greece; Thrombosis Research Laboratory, Department of Cardio-Thoracic-Vascular Sciences and Public Health, University of Padova, Padua, Italy; Autoimmune Diseases, Instituto de Investigación Sanitaria Biobizkaia, Barakaldo, Spain; Department of Obstetrics, University of Utah and Intermountain Healthcare, Salt Lake City, UT, USA; Clinical Rheumatology Unit, ASST G. Pini-CTO, University of Milan, Milan, Italy; Department of Clinical and Experimental Sciences, University of Brescia, Brescia, Italy; Servicio de Reumatologia, Hospital Universitario 12 de Octubre, Madrid, Spain; Rheumatology, Johns Hopkins University School of Medicine, Baltimore, MD, USA; Department of Autoimmune Diseases, Hospital Clínic, Universitat de Barcelona, Barcelona, Catalonia, Spain; Department of Rheumatology, Endocrinology and Nephrology, Faculty of Medicine and Graduate School of Medicine, Hokkaido University, Sapporo, Japan; Reumatologia, University of Sao Paulo, Sao Paulo, Brazil; Internal Medicine, University of Texas Medical Branch, Galveston, TX, USA; School of Cardiovascular & Metabolic Medicine & Sciences, King’s College London, London, UK; Barbara Volcker Center for Women and Rheumatic Disease, Hospital for Special Surgery, Weill Cornell Medicine, New York, NY, USA; Department of Haematology, University College London, London, UK; Department of Haematology, University College London Hospitals NHS Foundation Trust, London, UK; Department of Haematology, University College London, London, UK; Department of Haematology, University College London Hospitals NHS Foundation Trust, London, UK

**Keywords:** antiphospholipid syndrome, thrombosis, acquired protein C resistance, anti-protein C antibodies

## Abstract

**Objectives:**

The clinical significance of acquired resistance to activated protein C (APCr) and antibodies against protein C (anti-PCs) in APS has not been established. This study sought to determine the prevalence of APCr and anti-PCs, associations with aPL profile and clinical phenotypes, and exploratory associations with subsequent thrombosis.

**Methods:**

Three-hundred and seventy patients persistently positive for aPLs with/without APS (aPL-only, *n* = 77), obstetric APS (OAPS, *n* = 43), venous thromboembolism (VTE, *n* = 132), arterial thrombosis (AT, *n* = 87), or VTE + AT (*n* = 31) and 51 healthy controls were studied. Baseline APCr was determined using thrombin generation with recombinant human APC (rhAPC) or Protac^®^. Anti-PCs and avidity were detected by in-house ELISA.

**Results:**

All patient subgroups had markedly greater APCr compared with healthy controls (*P* < 0.001), with the greatest APCr observed in VTE + AT or triple aPL-positive patients. Anti-PCs were present in 42% of patients, with VTE + AT exhibiting significantly higher prevalence of high-avidity anti-PCs (79%) compared with aPL-only patients (38%) (*P* < 0.05). During prospective follow-up of 283 patients from the APS ACTION Registry (median 8.3 years), 25/283 (8.8%) had new thrombosis, with similar incidences between patients with or without APCr or anti-PCs (*P* > 0.60). Non-anticoagulated patients with APCr had more thrombotic events compared with those without, although the difference was not statistically significant (hazard ratio 4.5, 95% CI 0.8–25.9, *P* = 0.14).

**Conclusion:**

A high prevalence of APCr was seen across all aPL-positive patients, strongly associated with high-avidity anti-PCs. Lack of association with thrombosis may reflect confounding by baseline anticoagulation and limited events. The association of APCr with thrombosis in non-anticoagulated patients merits further exploration.

## Graphical abstract

**Graphical Abstract:**
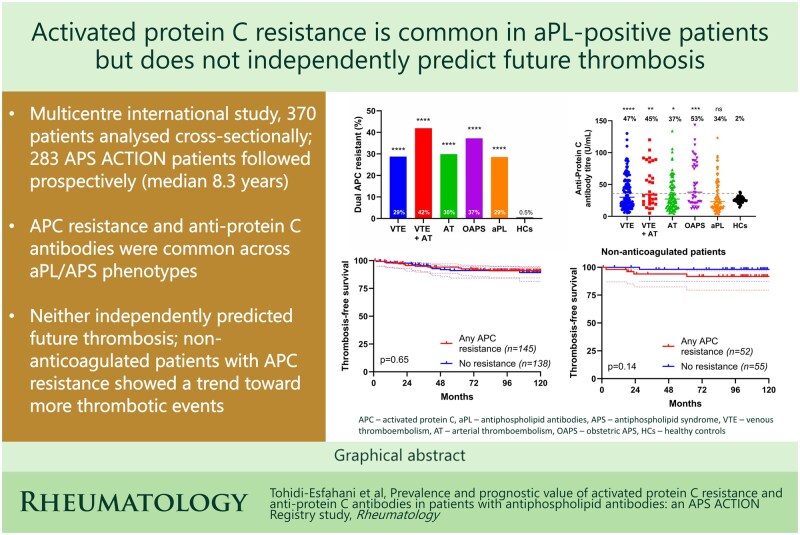

Rheumatology key messages

- Activated protein C resistance (APCr) and anti-protein C antibodies (anti-PCs) are common in APS.
- APCr and anti-PC do not independently predict future thrombosis in anticoagulated patients with APS.
- Non-anticoagulated patients with aPLs/APS and APCr showed a trend towards more thrombotic events.

## Introduction

APS is a heterogeneous autoimmune disorder characterized by persistence of aPLs, together with thrombosis and/or pregnancy morbidity [1]. The syndrome affects a relatively young patient population, with mean age at diagnosis 45 years for thrombotic APS [2]. Treatment failure occurs more often with standard-of-care therapy in APS; one study reported 44% of triple aPL-positive patients having recurrent thrombosis within 10 years [3]. While factors such as aPL profile and cardiovascular disease (CVD) risk factors have some prognostic value [4], good biomarkers for predicting treatment failure are needed.

Protein C is an endogenous anticoagulant that, together with its non-enzymatic cofactor protein S, prevents excess thrombin production by cleaving coagulation factors V (FV) and VIII following its proteolytic activation by thrombin–thrombomodulin [5]. Inherited severe deficiency of protein C manifests as life-threatening thrombosis in neonates [6]. Furthermore, activated protein C (APC) has anti-inflammatory and cytoprotective properties [7], thus making it an intriguing avenue of research in immune-related thrombosis disorders such as APS.

Interference with the protein C pathway has been reported in aPL-positive patients as a possible mechanism contributing to APS pathophysiology. Resistance to APC (APCr), commonly seen in FV Leiden mutations [8], is associated with increased thrombosis. Acquired APCr has long been identified in patients with APS [9]. Acquired APCr, assessed using the thrombin generation (TG) system, which provides a global assessment of coagulation function, with addition of APC, was shown to be associated with thrombosis in aPL-positive patients [10].

aPLs impair the cleavage of FV by APC [9, 11], with evidence of direct binding of β_2_ glycoprotein I (β_2_GPI) to APC in the presence of anti-β_2_GPI antibodies (aβ2GPI) contributing to APCr [12]. Other isotypes of aPL, including aCL and aPS-PTs [9, 13, 14], have also been implicated in APCr, with the potency of LA correlating with the degree of APCr [13]. Antibodies specific to protein C (anti-PC) have also been implicated in APCr in APS, with high-avidity anti-PCs associated with greater APCr [15]. Another mechanism implicated in APCr in APS is neutrophil extracellular traps (NETs), with a positive correlation with levels of NET markers and APCr [16].

Despite this growing literature on APCr in APS, it remains unclear whether APCr (or anti-PCs) can be utilized as a predictor of future thrombosis. Higher rates of APCr have been associated with historical thrombosis in several studies [10, 15, 17], with increased APCr and anti-PCs seen in patients with a history of greater thrombotic severity (anticoagulant-refractory thrombosis or both venous and arterial events) [15, 18]. A retrospective study associated anti-PCs with future thrombosis in SLE patients with or without aPL [19]; however, a small prospective study (*n* = 92) of patients with SLE or aPLs found no association of APCr with future thrombosis in multivariable analysis [20]. Another small prospective observational study suggested the highest centiles of APCr may predict future thrombosis, but this was established in only six patients in this group [21].

The lack of clarity around APCr being a predictive biomarker is likely driven by relatively small studies with heterogeneous patient groups and variable methodology. APCr is most commonly measured by TG assays with recombinant human APC (rhAPC) and/or Protac^®^, an enzyme isolated from snake venom, which activates endogenous protein C [22]. When APCr is tested with different modalities and protocols in APS patients, agreement is relatively poor and the results are not interchangeable [23].

The aim of this study was to determine the prevalence of APCr and anti-PCs in a large, well-characterized cohort of APS/aPL-positive patients, establish associations across clearly defined clinical phenotypes and aPL profiles, and assess exploratory associations of these two parameters with subsequent thrombosis in the prospectively followed subset of patients in The Antiphospholipid Syndrome Alliance for Clinical Trials and InternatiOnal Networking (APS ACTION) [24] clinical database and repository (‘registry’).

## Methods

### Study population and sample collection

A total of 370 patients were included [283 from APS ACTION and an additional 87 from the University College London Hospitals NHS Foundation Trust (UCLH)]. All 370 contributed to the baseline prevalence and cross-sectional phenotype analysis, with only the APS ACTION subset contributing to the prospective analysis. Patients recruited to the multicentre APS ACTION Registry had at least two tests in the prior 12 months demonstrating persistence of aPLs as per the revised Sapporo criteria [25]. Samples at entry to the registry were collected and used for this study. aPL results and APS-defining events prior to registry entry were entered retrospectively. Prospectively, patients had data entry with annual follow-up and/or at the time of subsequent thrombotic events. The study follow-up period was from time of entry to the registry to the first subsequent thrombosis or last follow-up.

Patients at UCLH were excluded if they had a factor V Leiden (*FVL*) mutation, current pregnancy or oral oestrogen use, active malignancy or myeloproliferative neoplasms, while APS ACTION eligibility followed the registry criteria [24]. Venous blood samples were collected into 0.105 M sodium citrate Vacutainer tubes (Becton Dickinson, Plymouth, UK), centrifuged twice (2000 *g*, 15 minutes) to obtain platelet-poor plasma, which was stored at −80°C for batch testing.

### aPL profiling

All baseline samples underwent testing at APS ACTION core laboratories following standardized assays and methodology. LA positivity was determined after performing screening, mixing and confirmatory testing with at least two different assays (dilute Russell Viper Venom Time and silica clot test or Taipan snake Venom/Ecarin Time dependent on anticoagulant), as per the International Society on Thrombosis and Haemostasis (ISTH) guidelines [26]. LA results were reported as positive or negative. Medium to high titres of aCLs and aβ2GPI were considered positive, as per the ISTH guidelines. Categorization of triple aPL positivity was limited to those with IgG antibodies.

### Activated protein C resistance measurement by thrombin generation assay

APCr was measured as previously described [15]. Briefly, the calibrated automated thrombinoscope (Diagnostica Stago, Maastricht, The Netherlands) platform was used for measuring TG, using the PPP-Reagent (Diagnostica Stago, 5pM tissue factor and 4 µM phospholipids). Samples from patients on vitamin K antagonist (warfarin) were mixed 1:1 with pooled normal plasma (PNP) to correct for coagulation factor deficiency, prior to testing. Resistance to exogenous APC was determined using rhAPC, and to activation of endogenous protein C using Protac^®^ (Pentapharm AG, Basel, Switzerland). APCr was expressed as percentage inhibition of endogenous thrombin potential (ETP), the total amount of thrombin generated over time, normalized to the results from PNP. The lower the percentage inhibition of ETP, the greater the APCr. Cut-off values for defining APCr were set at the 99th centiles of normal controls: rhAPC ≤ 56%, Protac^®^ ≤ 63%, as determined previously [19].

### Detection of anti-protein C antibodies and avidity determination

Anti-PCs and avidity were evaluated using in-house ELISA, as previously described [18]. Briefly, plates were coated with 10 μg/ml PC (Ceprotin, Baxter Healthcare Ltd, Norfolk, UK), with patient samples diluted 1:25 for testing. Standardized temperature and development time, and normalization using positive and negative controls between plates were used to standardize optical density values. The results were converted into U/ml, calculated in comparison with plasma from a patient with known high anti-PC levels, arbitrarily assigned as 100 U/ml. The cut-off for positivity was 36 U/ml, equivalent to greater than the 99th centile of normal controls. Non-specific binding was eliminated by subtracting the absorbance values from uncoated wells. Specificity of the assay for protein C has been previously established, with no cross-reactivity with β_2_GPI [18].

For avidity determination, all anti-PC–positive samples were diluted 100-fold in 0.15 M and 1.0 M concentrations of sodium chloride (NaCl) and then assayed for anti-PCs again. To differentiate high or low avidity, the initial binding at 0.15 M NaCl was compared with binding at 1 M NaCl, with avidity expressed as a percentage of maximum binding at 0.15 M NaCl (arbitrarily considered as 100%). High avidity was defined as >60% of the initial binding, based on previous studies [15].

Heritable thrombophilia testing was also performed with Protein C activity, free protein S antigen and antithrombin activity tested on a Sysmex CS‐2000*i* analyzer (Sysmex UK, Milton Keynes, UK), with reagents from Siemens Healthcare Diagnostics (Marburg, Germany). Factor V Leiden and prothrombin gene G20210A mutations were also evaluated where possible.

### Statistical analysis

Samples were analysed blinded and data analysis was performed using Graphpad Prism (La Jolla, California, USA). Continuous variables were compared between clinical groups using Kruskal–Wallis ANOVA with Dunn’s multiple comparisons test or Brown–Forsythe and Welch ANOVA/Dunnett’s multiple comparisons test, guided by testing of distribution normality. Categorical variable comparisons of proportions were performed using the χ^2^ test. A paired *t* test was used for paired results. Kaplan–Meier survival analysis was performed using the Log-rank (Mantel–Cox) test, with hazard ratio (HR) and 95% CI calculated (log-rank). Multivariable analysis was undertaken using multiple logistic regression (GraphPad Prism), with age, sex, clinical phenotype, antithrombotic use, triple aPL positivity, cardiovascular risk, and immunosuppression taken into account. Subgroup analyses were exploratory, and *P* values are nominal. A *P* value of <0.05 and a 95% CI not crossing 1.0 were considered statistically significant.

## Results

### Characteristics of the cohort at baseline

The baseline characteristics are summarized in Table 1. Patients were categorized by their clinical phenotype into five groups: aPL-positive without APS classification, obstetric APS (OAPS), venous thromboembolism only (VTE), arterial thromboembolism only (AT), or both venous and arterial thromboembolism (VTE + AT). Seventy-six patients (20.5%) were triple aPL-positive, with nearly 25% of the VTE or AT patients having experienced recurrent events before study entry. Baseline anticoagulation differed across subgroups, as did cardiovascular risk factors. Hypertension and hyperlipidaemia were more common in the AT group, whereas obesity was more frequent among those with a history of VTE. Around two-thirds of APS patients had primary APS, while more than half of the individuals in the aPL-only group had an associated autoimmune disease; SLE was present in 104 patients in total (28%), and was highest in the aPL-only cohort (49%). Overall rates of immunosuppressant use were similar across groups, although HCQ use was higher in the aPL-only and VTE + AT groups.

**Table 1:** Baseline characteristics of the cohorts.

Characteristics	aPL-only (*n* = 77)	Pregnancy morbidity (*n* = 43)	Venous thrombosis (*n* = 132)	Arterial thrombosis (*n* = 87)	Venous and arterial thrombosis (*n* = 31)
Age, mean, years ± s.d.	47.4 (±12.6)	42.6 (±8.7)	45.8 (±15)	48.8 (±13.2)	52.8 (±12.7)
Age, median, years (range, IQR)	46 (22–71, 37–58)	40 (24–65, 37–48)	44 (15–83, 35–55)	49 (24–80, 38.5–57)	58 (26–76, 40.3–61.8)
Sex, male/female, *n*	27/50	0/43	50/88	26/61	7/24
*FVL* mutation, *n* (%):					
Nil mutation	74 (96)	32 (74)	106 (80)	74 (85)	29 (94)
Unknown	3 (4)	11 (26)	26 (20)	13 (15)	2 (6)
Known inherited thrombophilia:					
Protein C deficiency	0 0	0 0	2 (2)	0 0	1 (3)
Protein S deficiency	0 0	1 (2)	3 (2)	0 0	0 0
Antithrombin deficiency	0 0	1 (2)	2 (2)	2 (2)	0 0
Prothrombin gene mutation^a^	4 (5)	5 (12)	4 (3)	3 (3)	2 (6)
aPL (IgG) status at baseline:	13 (17)	5 (12)	29 (22)	20 (23)	9 (29)
Triple positive, *n* (%)					
Single/double positive, *n* (%)	50 (65)	17 (40)	76 (58)	53 (61)	21 (68)
Known thrombosis risk factors, *n* (%):					
Obesity (BMI > 30)	18 (23)	5 (12)	44 (33)	10 (11)	10 (32)
DMell	3 (4)	3 (7)	8 (6)	6 (7)	2 (6)
HTN	19 (25)	6 (14)	27 (20)	34 (39)	14 (45)
Hyperlipidaemia	16 (21)	3 (7)	29 (22)	30 (34)	10 (32)
Smoking history	31 (41)	7 (16)	42 (32)	44 (51)	11 (35)
Autoimmune disease, *n* (%)	42 (54)	15 (35)	33 (25)	27 (31)	11 (35)
SLE-associated, *n* (%)	38 (49)	9 (21)	28 (21)	19 (22)	10 (32)
Recurrent thrombosis prior to baseline, *n* (%)	0 0	0 0	31 (23)	22 (25)	31 (100)
APS-related therapy^b^, *n* (%):					
Anticoagulation:					
Warfarin	11 (14)	3 (7)	100 (76)^c^	69 (79)	28 (90)
LMWH	0 0	2 (5)	2 (2)	3 (3)	0 0
Anti-Xa	0 0	0 0	18 (14)	0 0	2 (6)
Nil	66 (86)	38 (88)	12 (9)	13 (15)	1 (3)
Antiplatelet:					
Aspirin	55 (71)	16 (37)	14 (11)	40 (46)	9 (29)
Clopidogrel	1 (1)	1 (2)	1 (1)	5 (6)	3 (10)
Anticoagulant + antiplatelet	7 (9)	1 (2)	6 (4)	32 (37)	10 (32)
HCQ	48 (62)	13 (30)	48 (36)	32 (36)	16 (52)
Immunosuppression:					
CS	16 (21)	6 (14)	21 (16)	15 (17)	5 (16)
Rituximab	3 (4)	1 (2)	6 (5)	4 (5)	1 (3)
Mycophenolate	10 (13)	0 0	8 (6)	6 (7)	2 (6)
AZA	4 (5)	3 (7)	10 (8)	8 (9)	1 (3)

a All cases were heterozygous,

b HCQ and immunosuppression include current and previous use.

c 2 included as warfarin were on concurrent low-molecular-weight heparin (LMWH). aPL(-only): aPLs (without APS Classification); DMell: diabetes mellitus; FVL: Factor V Leiden; HTN: hypertension; IQR: interquartile range; LMWH: low-molecular-weight heparin.

### Prevalence of activated protein C resistance in patients with aPL or APS

**Figure 1:**
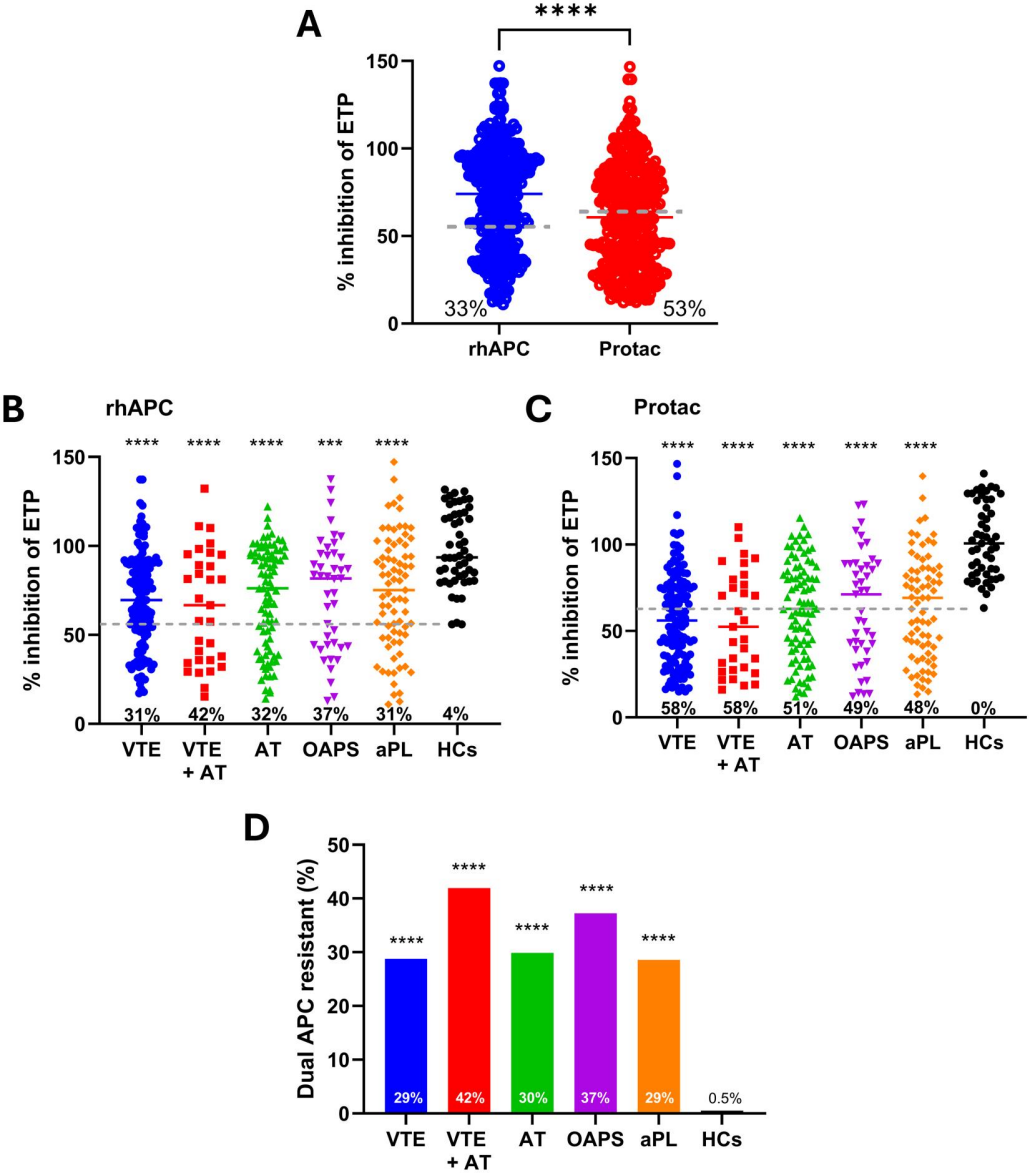
Patients with aPL have high rates of activated protein C (APC) resistance. Thrombin generation was measured on platelet-poor plasma collected from patients with aPL (*n* = 370) with or without clinical manifestations of APS and from healthy controls (HCs, *n* = 51); with endogenous thrombin potential (ETP) measured with recombinant human APC (rhAPC)/Protac^®^ or buffer. Percentage (%) inhibition of ETP was calculated after normalizing to ETP response to APC in pooled normal plasma. (A) Paired comparison of % inhibition of ETP by rhAPC and Protac^®^ in the patient cohort (*n* = 370). Patients were subcategorized into clinical manifestations of venous thromboembolism (VTE, *n* = 132), arterial thromboembolism (AT, *n* = 87), VTE + AT (*n* = 31), obstetric APS (OAPS, *n* = 43), carriers of aPL-only (aPL, *n* = 77) and compared with HCs (*n* = 51) with rhAPC (B) and Protac^®^ (C). Values on or below the grey dotted lines (56% for rhAPC, 63% for Protac^®^) are considered APC resistant, with percentages shown. The proportion of each clinical subcategory that were resistant to both rhAPC and Protac^®^ are shown (D), compared with HCs. ***P < 0.001, ****P < 0.0001

Overall, 55% of patients exhibited increased APCr. There was significantly higher prevalence of resistance to endogenous protein C with Protac^®^ (196/370, 53%) compared with rhAPC (33%, 122/370; *P* < 0.0001) in the overall patient cohort (Fig. 1A).

All cohorts exhibited significantly greater APCr to both rhAPC and Protac^®^, with a prevalence ranging from 31% to 42% and 48% to 58%, respectively, compared with healthy controls (*n* = 51, *P* < 0.001, Fig. 1B–C). Dual APCr (to rhAPC and Protac^®^) was detected in 31% (115/370) of patients, with the highest prevalence seen in patients with VTE + AT (42%) and OAPS (37%) (Fig. 1D). Pulmonary embolism (PE) was included within the VTE group, and APCr did not differ between PE and non-PE VTE. To determine whether the higher prevalence of APCr in VTE + AT was just a reflection of prior recurrent thrombotic events, patients within the VTE or AT cohorts who had recurrent VTE or recurrent AT (*n* = 53), were also assessed separately. Patients with historical VTE and AT still had a greater proportion of dual APCr, compared with patients with historical recurrent VTE or AT (dual APCr: Recurrent VTE or AT 35.8%, VTE + AT 42%, *P* = 0.58, Fig. S1). There was, however, a higher number of patients with only recurrent AT and dual APCr 50% (11/22) compared with patients with only recurrent VTE 25.8% (8/31) (*P* = 0.07), with numbers too small to draw firm conclusions. These results highlight the prevalence of APCr in APS, particularly in those with a history of VTE + AT.

### Prevalence of anti-protein C antibodies in patients with aPL or APS

**Figure 2:**
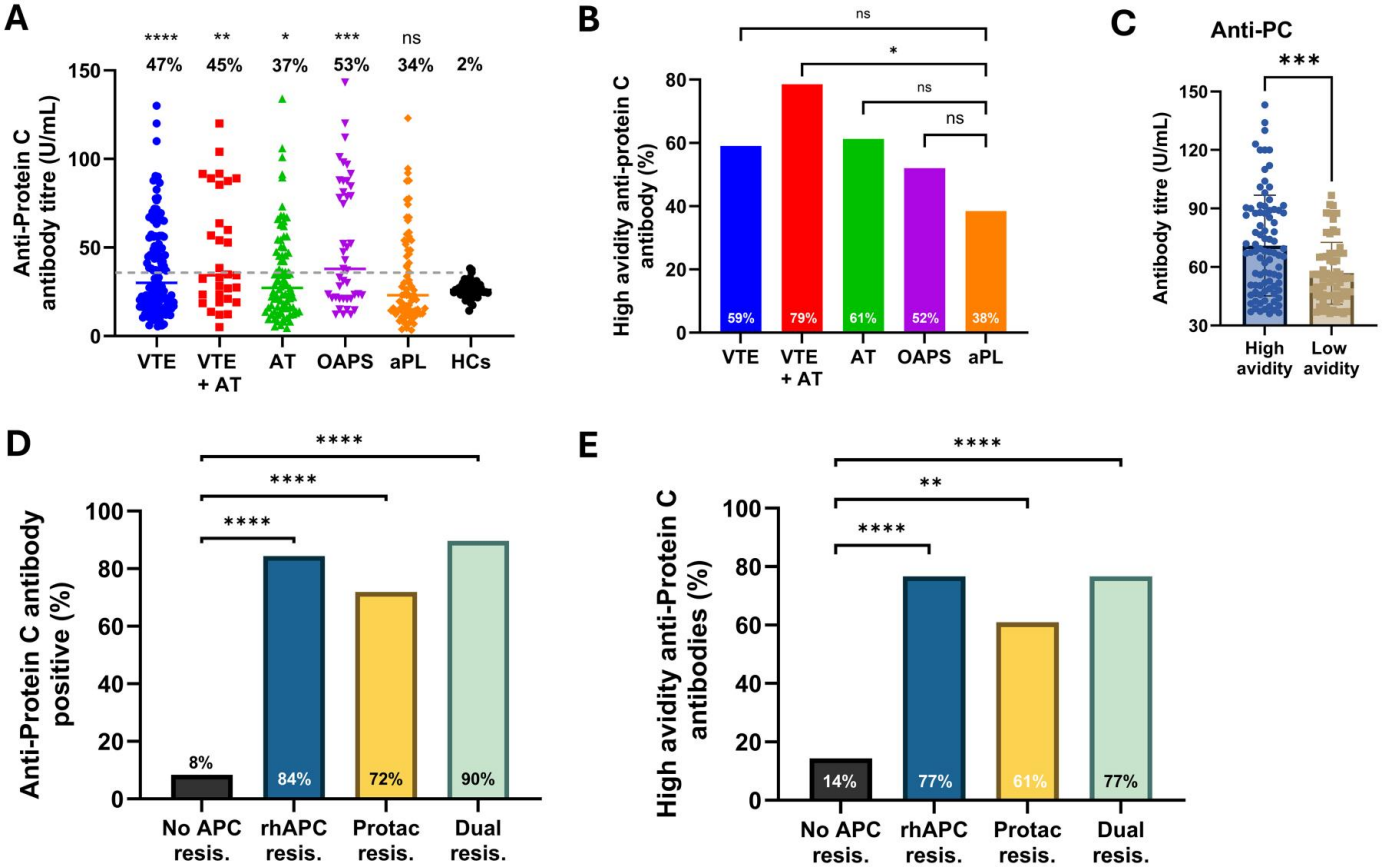
The majority of patients with activated protein C (APC) resistance have anti-protein C antibodies. Citrated plasma from APS patients with venous thromboembolism (VTE, *n* = 132), arterial thromboembolism (AT, *n* = 87), VTE + AT (*n* = 31), obstetric APS (OAPS, *n* = 43), carriers of aPL-only (aPL, *n* = 77) and healthy controls (HCs, *n* = 51) were diluted 1:25 and run on an in-house protein C ELISA to detect anti-protein C antibodies. (A) Antibody titres of the subjects in each group are shown, compared with HCs.. Values on or above the grey dotted line (36U/mL) are considered positive for anti-protein C antibody, with the percentage of each group positive for antibody shown. (B) The proportion of the antibodies detected (VTE *n* = 61, VTE + AT *n* = 14, AT *n* = 31, OAPS *n* = 23, aPL *n* = 26) that were high avidity were compared with that of the aPL-only cohort. (C) The antibody titres of all high-avidity (*n* = 88) and low-avidity (*n* = 66) anti-protein C antibodies were compared. (D) The proportion of patients with no APC resistance (*n* = 167), recombinant human APC (rhAPC) resistance (*n* = 122), Protac^®^ resistance (*n* = 196) and resistance to both rhAPC and Protac^®^ (*n* = 115) with anti-protein C antibodies. (E) The proportion of anti-protein C antibodies that had high avidity in each of the APC resistance groups. ns: not significant; resis.: resistance. *P < 0.05, **P < 0.01, ***P < 0.001, ****P < 0.000

Forty-two percent (156/370) of all patients were positive for anti-PCs. Anti-PCs were detected in all patient cohorts, with the highest prevalence in the OAPS, VTE and VTE + AT cohorts: 53%, 47% and 45%, respectively, and the lowest prevalence in aPL-only patients (34%) (Fig. 2A). All patients appeared to have high rates of high-avidity anti-PC, although only the VTE + AT group had a significantly higher prevalence (79%) compared with aPL-only patients (38%)(*P* < 0.05, Fig. 2B). While there was significant overlap, the anti-PC titre was significantly higher with high-avidity anti-PC than with low-avidity antibodies (Fig. 2C).

There was a strong association between increased APCr and presence of anti-PCs, especially in patients with dual APCr: 89.6% had anti-PCs (Fig. 2D). The anti-PC titre had a strong negative correlation with % ETP inhibition for both rhAPC and Protac^®^ (*P* < 0.0001, Fig. S2). Anti-PCs were predominantly high avidity in those with APCr: 77% in patients with dual APCr (*n* = 103, Fig. 2E). Comparatively, only 14% of the anti-PCs (2 of 14) detected in patients with no APCr were high avidity (*P* < 0.0001). These results are consistent with a contributory role for anti-PCs, most commonly high-avidity, in increased APCr.

### Activated protein C resistance and anti-protein C antibodies in triple-aPL, LA-positive and SLE patients with aPL

**Figure 3:**
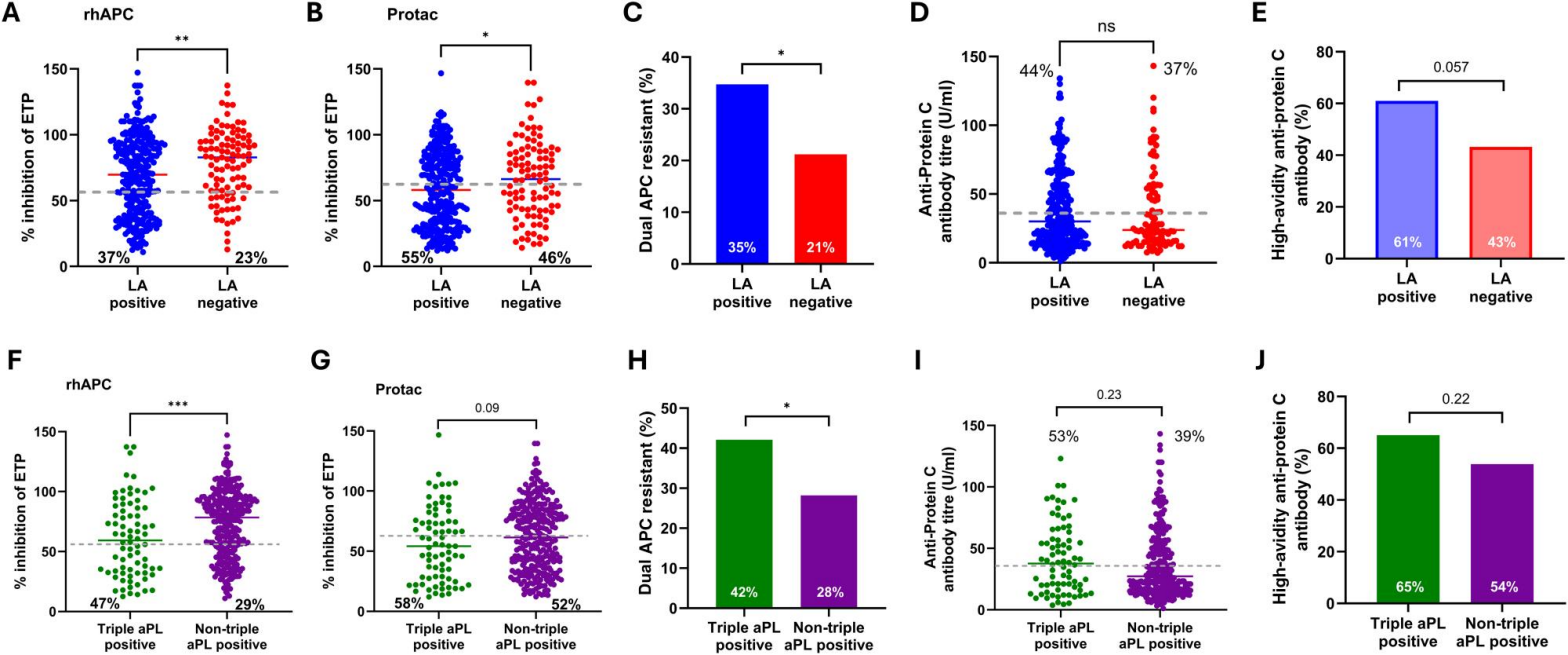
Higher-risk aPL profiles have greater activated protein C (APC) resistance. LA-positive patients (*n* = 271) were compared with LA-negative patients (*n* = 99) with regards to APC resistance to recombinant human APC (rhAPC) (A), Protac^®^ (B), or resistance to both (C), measured by thrombin generation assay on citrated plasma, as well as proportion of anti-protein C antibodies (D) and proportion of anti-protein C antibodies that are high avidity (E) by in-house ELISA. The same comparisons were performed for triple aPL-positive (IgG subtype only) patients (*n* = 76) and non-triple aPL-positive patients (*n* = 294) (F–J). Values on or below the grey dotted lines (56% for rhAPC, 63% for Protac^®^) are considered APC resistant, and values on or above the grey dotted line (36 U/mL) are considered positive for anti-protein C antibody. ns: not significant; ETP: endogenous thrombin potential. *P < 0.05, ***P < 0.001

Patients positive for LA at baseline (*n* = 271) had significantly greater APCr (rhAPC, Protac^®^ and dual APCr) compared with those negative for LA (*n* = 99) (*P* < 0.05, Fig. 3A–C). A higher number of LA-positive compared with LA-negative patients had anti-PCs (44% *vs* 37%); however, there was no difference in the overall titres: mean anti-PC titre LA-positive 38.2 U/ml, LA-negative 38.3 U/ml (*P* = 0.9, Fig. 3D). LA-positive patients with anti-PCs showed a trend towards association with high-avidity antibodies: LA-positive 61% high avidity, LA-negative 43.2%, *P* = 0.06 (Fig. 3E).

Further analysis according to aPL profile revealed that triple (IgG) aPL-positive patients had significantly greater APCr to rhAPC when compared with non-triple aPL-positive patients (*P* = 0.0009, Fig. 3F); however, the difference in APCr to Protac^®^ did not reach statistical significance (*P* = 0.09, Fig. 3G). Notably, there was a higher percentage of triple aPL-positive patients (42.1%) with dual APCr compared with patients with other aPL profiles (28.2%, *P* = 0.02, Fig. 3H). There was a trend towards higher prevalence and avidity of anti-PCs in triple aPL-positive patients (Fig. 3I, J).

The impact of SLE on the protein C axis was also assessed. There was no difference in APCr, presence of anti-PCs, or anti-PC avidity in aPL-positive/APS patients with or without SLE (Fig. S3).

### Thrombosis-free survival in APS/aPL-only clinical subgroups

As shown previously [15], and confirmed here, there is increased association between APCr and higher-risk APS patients, namely APS patients with VTE + AT or triple aPL positivity. Out of the 370 patients, 283 patients (including aPL-only and APS) were prospectively followed for a median of 99 (interquartile range 72–115) months, with subsequent thrombosis occurring in 8.8% (25/283). Where thrombosis type was known (*n* = 24), 18 had AT and 9 VTE; 3 patients experienced both AT and VTE during follow-up. Patients with prior VTE + AT had the worst thrombosis-free survival rates, even when including patients with prior recurrent VTE or recurrent AT in the comparison (*P* = 0.03, Fig. S4A): estimated thrombosis-free survival at 8 years for aPL-only (*n* = 77) was 95%, OAPS (*n* = 22) 95%, AT (*n* = 46) 95%, VTE (*n* = 58) 93%, recurrent VTE or AT (*n* = 53) 85%, and VTE + AT (*n* = 27) 78% (Fig. S4A). Triple IgG aPL-positive patients (at baseline) had lower, but not significant (*P* = 0.36), thrombosis-free survival, estimated 87% at 8 years compared with non-triple aPL-positive patients (92%) (Fig. S4B). Given historical VTE + AT and baseline triple aPL positivity were associated with greater APCr, these poorer outcomes suggest a possible association between APCr and high-risk patients.

### Utility of activated protein C resistance and anti-PC to predict future thrombosis in patients with aPLs or APS

**Figure 4:**
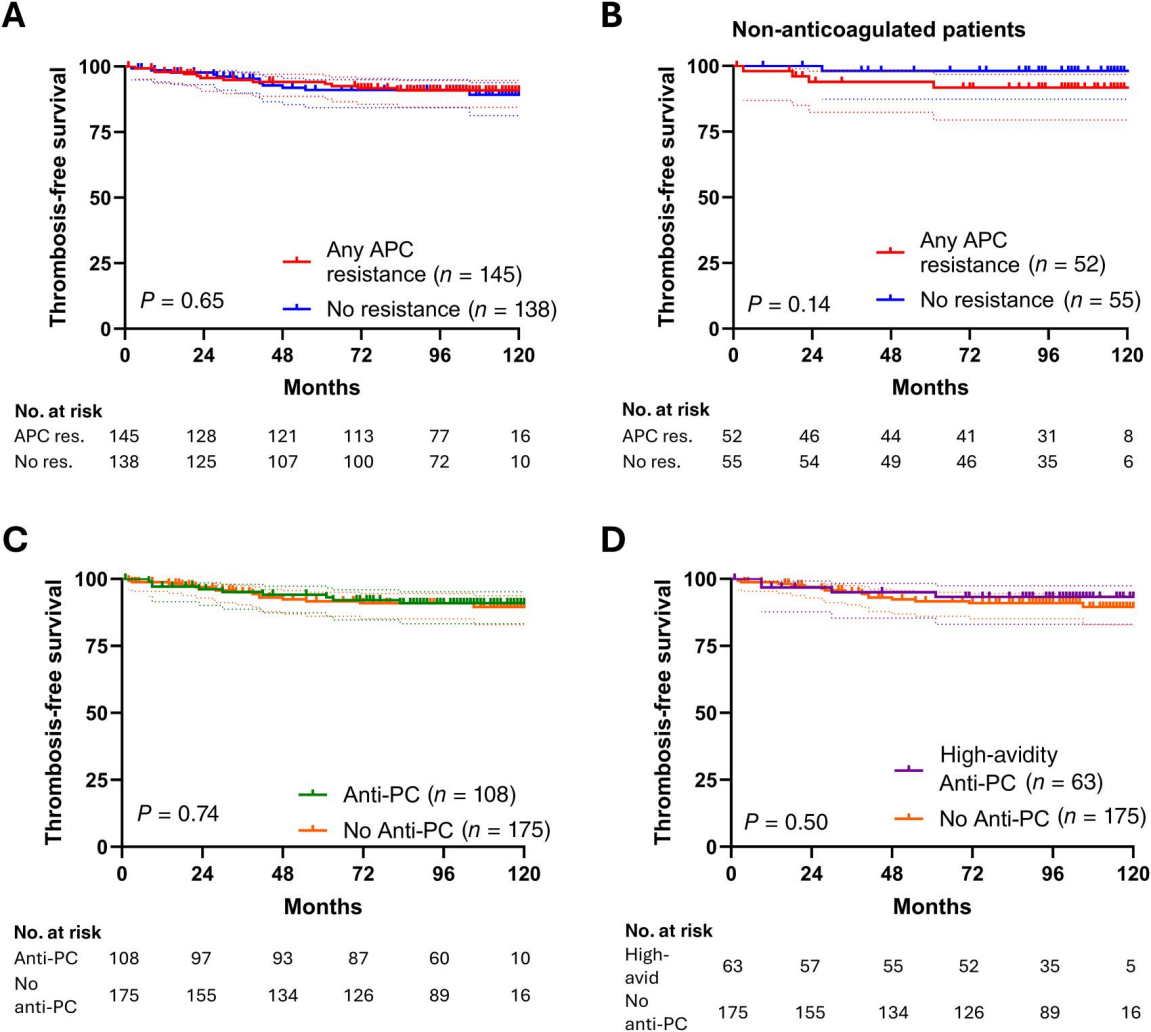
Baseline activated protein C resistance and anti-protein C antibodies do not predict future thrombosis in aPL-positive patients. Kaplan–Meier thrombosis-free survival analysis of prospectively followed patients within the APS alliance for clinical trials and international networking (APS ACTION) registry with or without baseline resistance to either recombinant human APC or Protac^®^ in a thrombin generation assay (A) or limited to patients not anticoagulated at time of baseline sample (B). The same analysis was performed comparing patients with or without anti-protein C antibodies (anti-PC) (C) and those with high-avidity anti-PC to those without anti-PC (D). The dotted lines represent the 95% CIs. No. at risk: number at risk (those that are remaining without event or being censored)

Despite being more prevalent in patients with a more severe phenotype (VTE + AT) and in triple-aPL-positive patients, APCr at baseline (entry to APS ACTION registry, not at diagnosis) had no predictive value for future thrombosis. Patients with APCr to either rhAPC or endogenous protein C (Protac^®^) at baseline (*n* = 145) had the same rates of thrombosis as those without (*n* = 138, *P* = 0.60, Fig. 4A). The same held true when comparing patients with APCr greater than the 90th centile (*n* = 28) with those with APCr below the 90th centile (Fig. S5), and after multivariable analysis, where CVD risk factors and prior VTE + AT were predictive (Table 2). There was, however, a trend towards an association between new thrombotic events and APCr observed in aPL-only and APS patients on no anticoagulation at baseline (hazard ratio 4.5, 95% CI 0.8–25.9, *P* = 0.14, Fig. 4B), with an odds ratio of 7.27 (CI 0.8–168.1, *P* = 0.08, Table 2) in multivariable analysis. Given the small number of events, these estimates can only be hypothesis generating. Similar to APCr, the presence of anti-PCs and anti-PC avidity also did not predict future thrombosis (Fig. 4C, D, Table S1). Patients with subsequent thrombotic events during retrospective and prospective follow-up (*n* = 30) did not have greater APCr or positivity of anti-PCs at baseline (Fig. S6). The proportion of anti-PCs that were high avidity also did not differ in those with or without subsequent thrombosis: 8/13 (62%) in those with subsequent thrombosis and 80/142 (56%) in patients without subsequent thrombosis were high avidity (*P* = 0.72).

**Table 2:** Multivariable analysis for baseline predictors of future thrombosis in the Prospective APS ACTION total cohort (*n* = 283)

**Prospective APS ACTION total cohort (*n* = 283)**			
Variable	Odds ratio	95% CI	*P*-value
**Age**	**0.96**	**0.92–0.999**	**0.045**
Sex—female	0.38	0.14–1.02	0.054
APCr^a^	0.67	0.27–1.64	0.38
Triple aPL positivity^b^	0.77	0.25–2.18	0.63
**Cardiovascular risk factors^c^**	**3.90**	**1.37–12.20**	**0.01**
Obesity	0.54	0.16–1.60	0.28
APS subgroup^d^			
Prior VTE	2.91	0.80–12.79	0.11
Prior AT	0.97	0.18–5.23	0.97
**Prior VTE and AT**	**9.33**	**1.95–51.46**	**0.005**
Obstetric APS without prior thrombosis	1.72	0.08–15.21	0.69
SLE	1.44	0.48–4.34	0.51
Anticoagulant and antiplatelet	1.63	0.50–5.07	0.41
HCQ	0.71	0.26–1.84	0.48
Immunosuppression	0.57	0.17–1.73	0.33
***Prospective APS ACTION cohort non-anticoagulated at baseline (n = 107)***			
Variable	Odds ratio	95% CI	*P*-value
Age	0.94	0.84–1.03	0.21
Sex—female	0.78	0.05–21.16	0.86
APCr^a^	7.27	0.82–168.1	0.08
Triple aPL positivity^b^	0.31	0.01–3.34	0.35
Cardiovascular disease risk factors^c^	1.23	0.10–11.38	0.86
Antiplatelet	0.27	0.03–2.50	0.23
HCQ	0.14	0.01–1.18	0.07

a APCr–either resistance to activated protein C (APC) by recombinant human APC or Protac^®^.

b IgG-subtype only,

c Hypertension, hyperlipidaemia, diabetes and/or smoking,

d Reference level for analysis of clinical subgroups was aPL-only cohort. AT: arterial thromboembolism; VTE: venous thromboembolism.

Variables in bold text are those that achieved statistical significance (*P* < 0.05).

The presence of APCr was also not differentiating within aPL profile subgroups, including patients with LA positivity alone and triple aPL positivity (Fig. S7A–B), nor in patients with SLE (Fig. S7C). Within clinical subgroups, there was a trend towards APCr being predictive of future thrombosis in patients with OAPS or aPL-only (hazard ratio 8.3, CI 0.2–418.4, *P* = 0.29 and hazard ratio 2.9, CI 0.4–20.6, *P* = 0.29, respectively, Fig. S8), reflecting the trend seen in non-anticoagulated patients.

## Discussion

This study presents the largest prospectively followed cohort of aPL-positive patients assessing the prevalence of APCr (to rhAPC and to activation of endogenous protein C by Protac^®^) and anti-PCs; and whether the two parameters have any association with subsequent thrombosis. The study identified a high prevalence of APCr in patients with aPLs and a clear correlation between APCr and high-avidity anti-PCs with high-risk disease, confirming previous observations in a larger well-characterized cohort. Despite neither APCr nor anti-PC being predictive of thrombosis in anticoagulated patients, there was a signal that APCr might identify non-anticoagulated patients more likely to have thrombosis.

This research highlights and further supports a strong link between aPL positivity and APCr, particularly in patients with LA or triple aPL positivity. This is reflected in the literature previously showing that different aPL subtypes can impair APC activity in cleaving FV, with antibody titre, number and LA potency positively correlating with APCr [9, 11–13, 27]. In this study, resistance to activation of endogenous protein C was more frequent than resistance to exogenous APC in all aPL-positive subgroups, suggesting a possible defect in the mechanism of activation of protein C rather than interference with the action of APC and the proteolytic inactivation of FV. β2GPI has been shown to selectively inhibit the procoagulant functions of thrombin, but not its ability to activate protein C [28], further providing mechanistic evidence for the prothrombotic consequences of APCr in APS. Anti-PCs also appear to have a large role in driving the APCr, with almost all patients with anti-PCs having APCr (92%). Given we have previously shown the anti-PCs in this assay are specific and do not cross-react with β_2_GPI [18], this correlation with APCr suggests an aPL-independent effect. Yet, another study has shown some cross-reactivity between aβ2GPI and APC [29], suggesting that some aPLs could target shared epitopes, contributing to the APCr.

This study confirmed the association of APCr and high-risk patients [15], especially patients with VTE + AT, who had the highest recurrent thrombosis rates in this prospective study. There was also a signal of higher APCr in APS patients with recurrent AT compared with recurrent VTE, although the numbers were too small to draw firm conclusions. The question remains as to whether the APCr is a parallel or driver phenomenon of the increased risk. Previously, a prospective study by Zuily *et al.* [21] found that patients with greater than the 95th centile APCr had a hazard ratio of 6.1 (95% CI 1.7–21.9) for thrombosis recurrence. However, this significance was achieved with three events in six patients, the small numbers limiting firm conclusions. In this study, thrombosis rates within the entire cohort were similar in patients with or without APCr or anti-PCs, suggesting APCr may be a parallel phenomenon. Yet, non-anticoagulated patients with APCr had a hazard ratio of 4.5 for thrombosis (95% CI 0.8–25.9), with a strengthened association seen with multivariable analysis. This suggests APCr could be increasing risk of thrombosis in APS, a risk possibly ameliorated by anticoagulation.

The study’s limitations include prospective follow-up occurring from entry into the registry, rather than from diagnosis. Obstetric outcomes were outside this study’s scope, and therefore prospective analysis focused on thrombosis only. There were 15% of patients with unknown *FVL* mutation status, although this was only 3% in the prospectively followed cohort, and none of whom had APCr. In addition, the study was not designed to analyse the predictors of future thrombosis in a multivariable fashion, including other clinical and biologic markers. Due to the low number of new thrombotic events (*n* = 25), CIs were wide. As such, absence of significance does not exclude clinically relevant effects. Our results may be indicative of the different pathophysiology driving thrombosis in APS, which can be very heterogeneous, involving pathways separate to coagulation dysregulation, e.g. platelet hyperreactivity [30]. Assessing APCr in the context of other pathophysiological biomarkers will allow for better delineating of its role in APS-related thrombosis, potentially guiding tailored therapy to improve the outcomes seen with anticoagulation alone.

In conclusion, APCr is highly prevalent in persistently aPL-positive patients, with the highest prevalence in ‘higher risk’ APS patients. This study of a large, well-defined cohort prospectively followed for a median of over 8 years did not identify APCr or anti-PC as predictive biomarkers for future thrombosis in anticoagulated patients. The findings of a trend towards increased thrombosis in non-anticoagulated patients with APCr supports prospective evaluation of APCr for risk stratification in non-anticoagulated aPL-positive individuals. Identifying and navigating the heterogeneous pathophysiological pathways in APS will facilitate more targeted, patient-tailored therapy.

The participating APS ACTION centres and UCLH each had ethics board approval, with written informed consent obtained from each participant, in accordance with the Declaration of Helsinki. Ethical approval was granted by the Research Ethics Committee (NREC reference: 13/NI/0049) and from the Research and Development office at UCLH (reference: 13/0030).

## Supplementary Material

keag304_Supplementary_Data

## Data Availability

To request access to the study data, please contact the corresponding author.
