# Supplementary material for: Prevalence and prognostic value of activated protein C resistance and anti–protein C antibodies in patients with aPLs: an APS ACTION Registry study
Source: Rheumatology (Oxford). 2026 Jun 13;65(7):keag304. doi: 10.1093/rheumatology/keag304 (PMC13344845; doi:10.1093/rheumatology/keag304)
Supplement: keag304_Supplementary_Data [file keag304_supplementary_data.zip › 01-Jul-2026_074423_rhe-26-0428-File007.docx]

**Supplementary Materials for Manuscript by Tohidi-Esfahani et al: *Prevalence and prognostic value of activated protein C resistance and anti-protein C antibodies in patients with antiphospholipid antibodies: an APS ACTION Registry study***

**Supplementary Tables**

**Table S1**

| ***Table S1. Multivariable analysis for baseline predictors of future thrombosis (Anti-PC)***  ***Prospective APS ACTION total cohort (n=283)*** | | | |
| --- | --- | --- | --- |
| Variable | Odds Ratio | 95% CI | P-value |
| **Age** | **0.96** | **0.92 – 0.999** | **0.045** |
| Sex – Female | 0.37 | 0.14 – 0.998 | 0.050 |
| Anti-PC | 0.79 | 0.30 – 1.96 | 0.62 |
| Triple aPL positivity^#^ | 0.77 | 0.25 – 2.17 | 0.63 |
| **Cardiovascular risk factors^** | **3.67** | **1.30 – 11.39** | **0.01** |
| Obesity | 0.57 | 0.17 – 1.67 | 0.31 |
| APS subgroup^^ |  |  |  |
| Prior VTE | 2.90 | 0.80 – 12.63 | 0.11 |
| Prior AT | 1.03 | 0.20 – 5.45 | 0.98 |
| **Prior VTE & AT** | **9.65** | **2.01 – 53.45** | **0.005** |
| PM without prior thrombosis | 1.85 | 0.09 – 16.29 | 0.63 |
| Systemic Lupus Erythematosus | 1.47 | 0.49 – 4.42 | 0.49 |
| Anticoagulant and antiplatelet | 1.55 | 0.48 – 4.78 | 0.46 |
| Hydroxychloroquine | 0.72 | 0.26 – 1.87 | 0.50 |
| Immunosuppression | 0.59 | 0.18 – 1.78 | 0.36 |

^#^IgG-subtype only, ^Hypertension, hyperlipidaemia, diabetes and/or smoking, ^^Reference level for analysis of clinical subgroups was aPL-only cohort. Anti-PC – antibodies to protein C, aPL – antiphospholipid antibodies, APS – antiphospholipid syndrome, VTE – venous thromboembolism, AT – arterial thromboembolism

**Supplementary Figures**


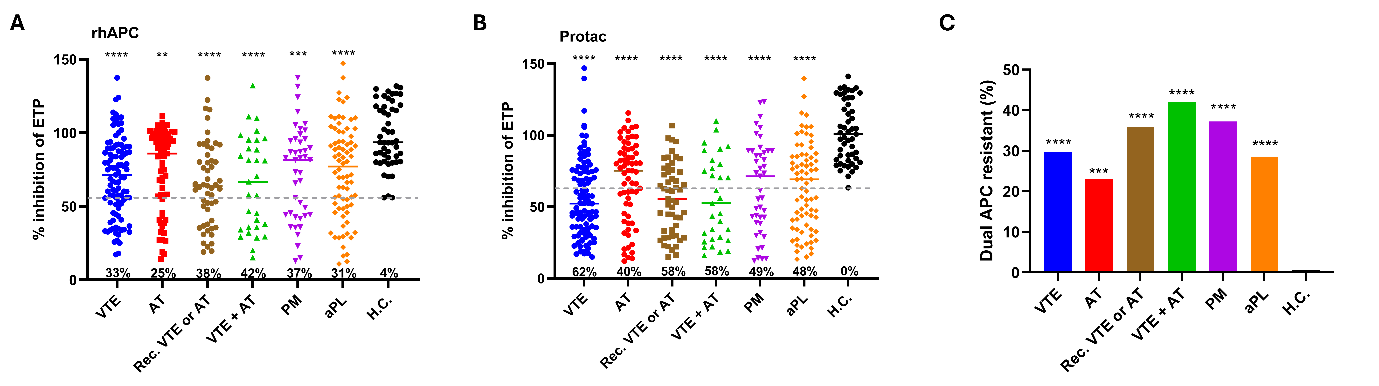
**Supplementary** **Figure S1. Patients with antiphospholipid antibodies (aPL) and both venous and arterial thrombosis have the highest rates of activated protein C (APC) resistance.** Thrombin generation was measured on platelet-poor plasma collected from patients with aPL (n=370) with or without clinical manifestations of antiphospholipid syndrome and healthy controls (H.C., n=51); with endogenous thrombin potential (ETP) measured with recombinant human APC (rhAPC)/Protac or buffer. Percent (%) inhibition of ETP was calculated after normalising to ETP response to APC in pooled normal plasma. Patients were subcategorised into clinical manifestations of venous thromboembolism (VTE, n=101), arterial thromboembolism (AT, n=65), recurrent VTE or recurrent AT prior to baseline (Rec. VTE or AT, n=53), VTE + AT (n=31), pregnancy morbidity (PM, n=43), carriers of aPL-only (aPL, n=77) and compared to H.C. (n=51) with rhAPC (A) and Protac (B). Values on or below the grey dotted lines (56% for rhAPC, 63% for Protac) are considered APC resistant, with percentages shown. The proportion of each clinical subcategory that were resistant to both rhAPC and Protac are shown (C), compared to H.C. ***p<0.001, ****p<0.0001


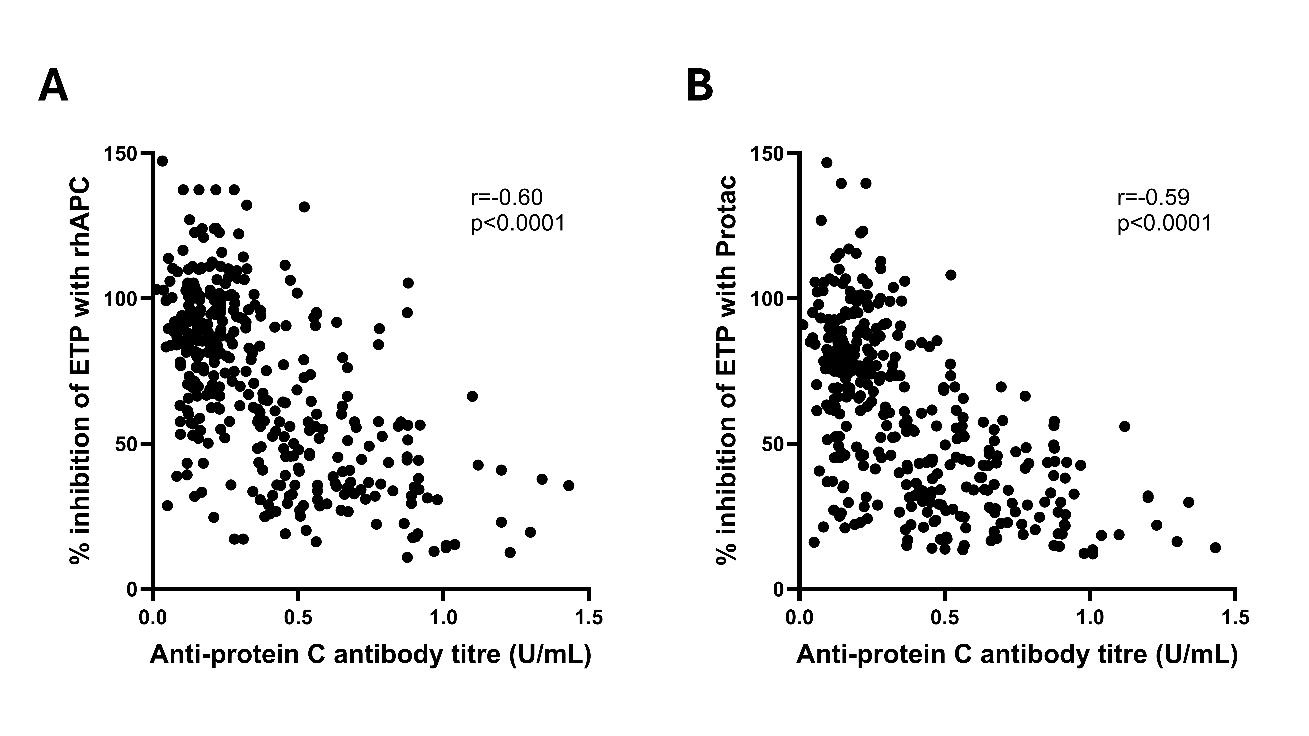


**Supplementary Figure S2. Anti-protein C antibody titres correlate with degree of activated protein C resistance.** Percent (%) inhibition of Endogenous thrombin potential (ETP) measured in the thrombin generation assay with either recombinant human APC (rhAPC, A), or Protac® (B), was correlated with anti-protein C antibody titre (n=370).


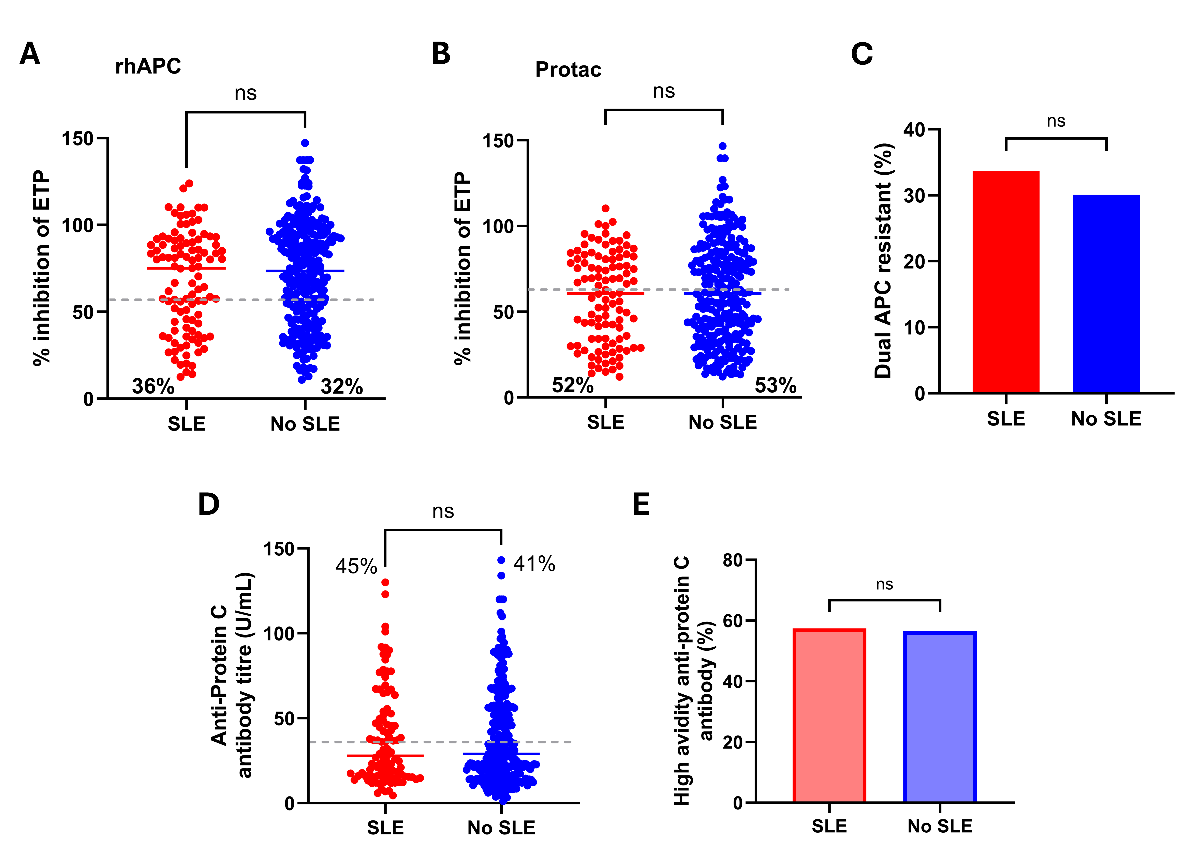


**Supplementary Figure S3. Systemic lupus erythematosus patients (SLE) with antiphospholipid antibodies (aPL) have similar activated protein C (APC) resistance to non-SLE patients.** Patients with aPL and SLE (n=104) were compared to patients with aPL and no SLE (n=266) with regards to APC resistance to recombinant human APC (rhAPC) (A), Protac (B), or resistance to both (C), measured by thrombin generation assay on citrated plasma, as well as proportion of anti-protein C antibodies (D) and proportion of anti-protein C antibodies that are high avidity (E) by in-house ELISA. Values on or below the grey dotted lines (56% for rhAPC, 63% for Protac) are considered APC resistant, and values on or above the grey dotted line (36U/mL) are considered positive for anti-protein C antibody. ns – not significant, ETP – endogenous thrombin potential


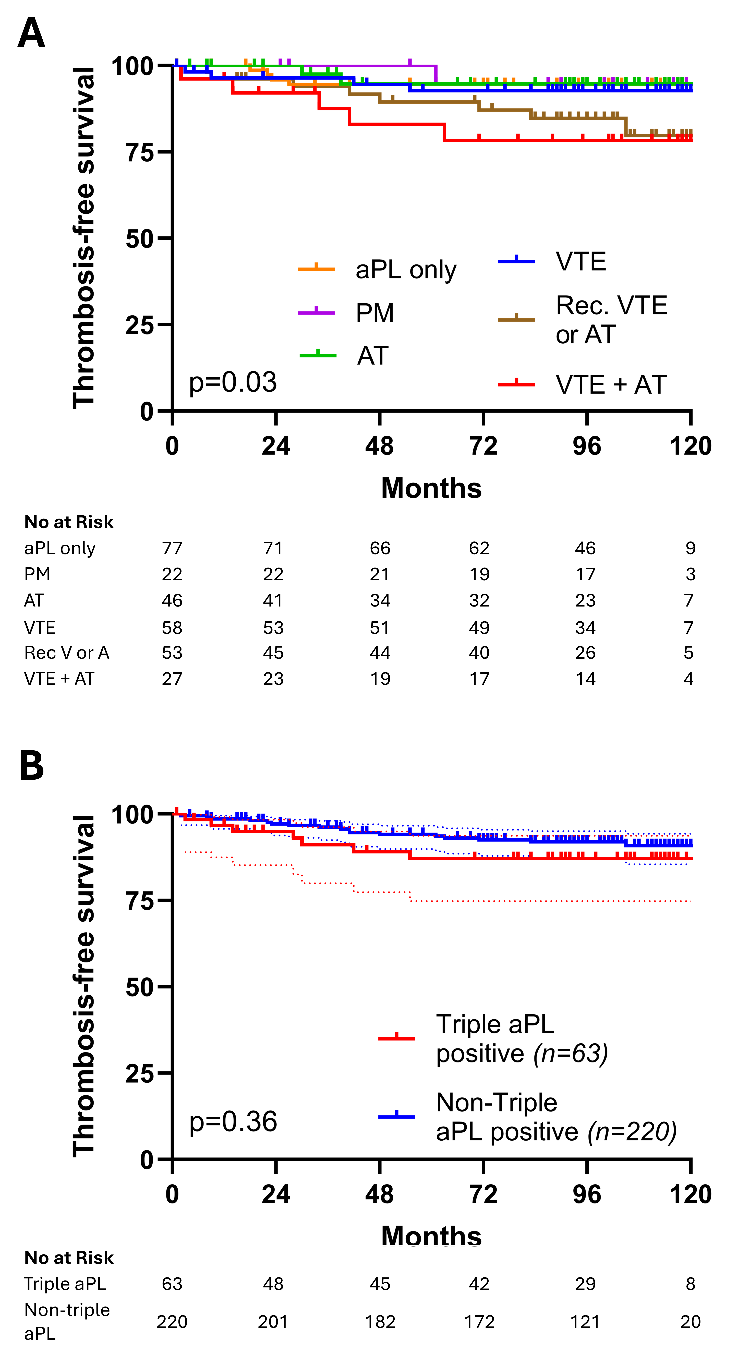


**Supplementary Figure S4. Thrombosis-free survival rates in patients within clinical subgroups of patients with antiphospholipid antibodies (aPL).** (A) Kaplan-Meier thrombosis-free survival analysis of prospectively followed patients with aPL-only (n=77), history of single venous thromboembolism event (VTE, n=58), single arterial thromboembolism event (AT, n=46), history of recurrent VTE or recurrent AT (Rec. VTE or AT, n=53), history of both VTE and AT (VTE + AT, n=27) and pregnancy morbidity (PM, n=22). Kaplan-Meier thrombosis-free survival analysis of prospectively followed triple aPL positive (n=63) and non-triple aPL positive patients (n=220) (B). No at risk – number at risk (those that are remaining without event or being censored), Rec V or A - recurrent VTE or recurrent AT


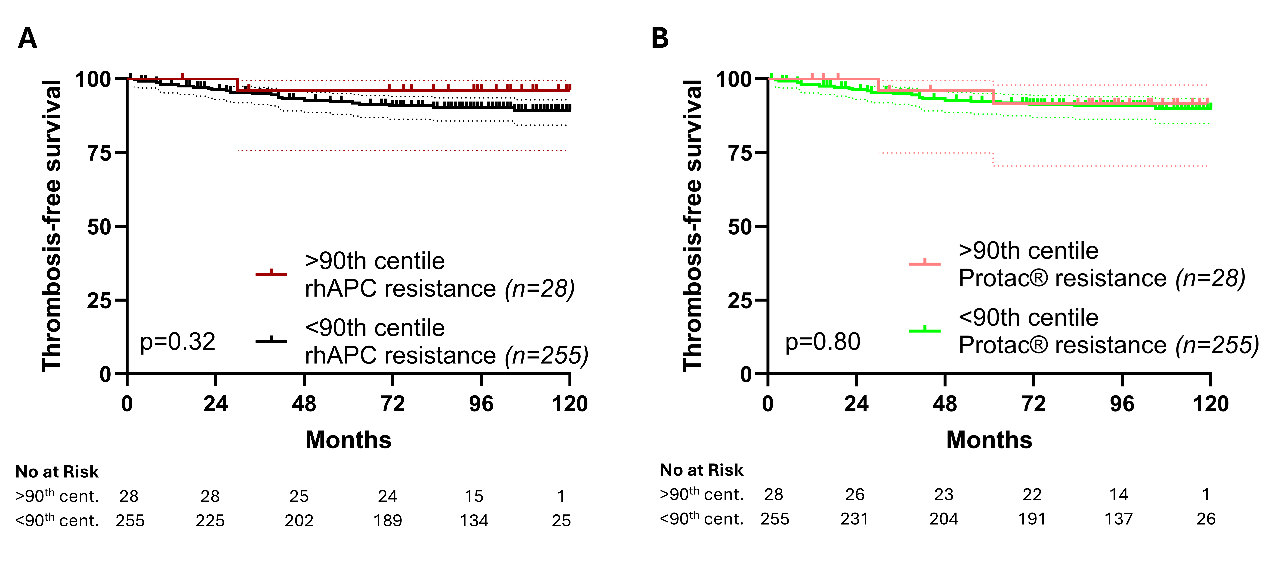


**Supplementary Figure S5. Antiphospholipid antibody positive patients with the highest degrees of activated protein C (APC) resistance do not have increased future thrombosis.** Kaplan-Meier thrombosis-free survival analysis of prospectively followed patients within the antiphospholipid syndrome alliance for clinical trials and international networking (APS ACTION) registry with >90^th^ centile resistance to either recombinant human APC (A) or Protac (B) compared to those with <90^th^ centile resistance in a thrombin generation assay. The dotted lines represent the 95% confidence intervals. No at risk – number at risk (those that are remaining without event or being censored)


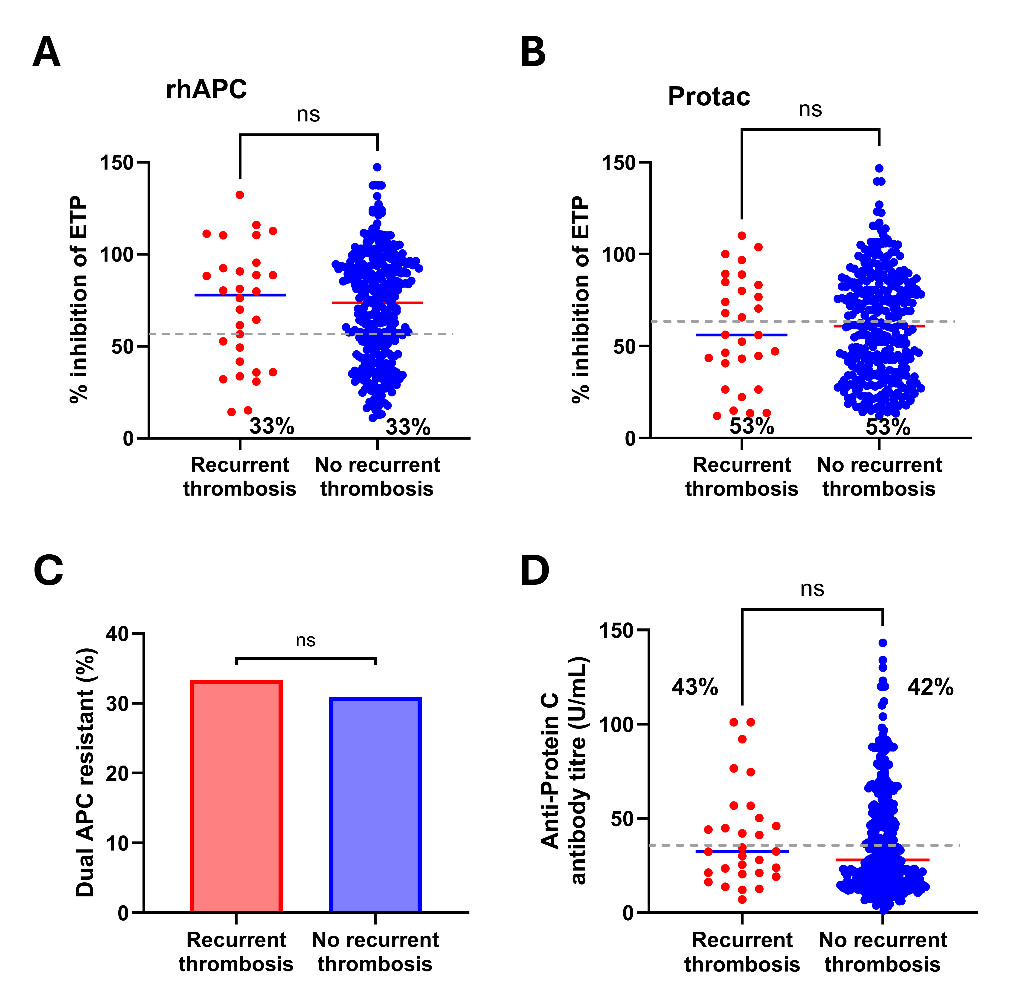


**Supplementary Figure S6. Patients with thrombosis during follow up did not have increased activated protein C (APC) resistance or anti-protein C antibodies at baseline.** Patients who had subsequent thrombosis during prospective and retrospective follow up (n=30) were compared to those that did not (n=340). Thrombin generation was measured on platelet-poor plasma collected from patients with endogenous thrombin potential (ETP) measured with or without recombinant human APC (rhAPC) (A) or Protac (B). Percent (%) inhibition of ETP was calculated after normalising to ETP response to APC in pooled normal plasma. Values on or below the grey dotted lines (56% for rhAPC, 63% for Protac) are considered APC resistant. Rates of resistance to both rhAPC and Protac were also calculated (C). The proportion of anti-protein C antibodies were measured by in-house ELISA (D). Values on or above the grey dotted line (36U/mL) are considered positive for anti-protein C antibody. ns – not significant


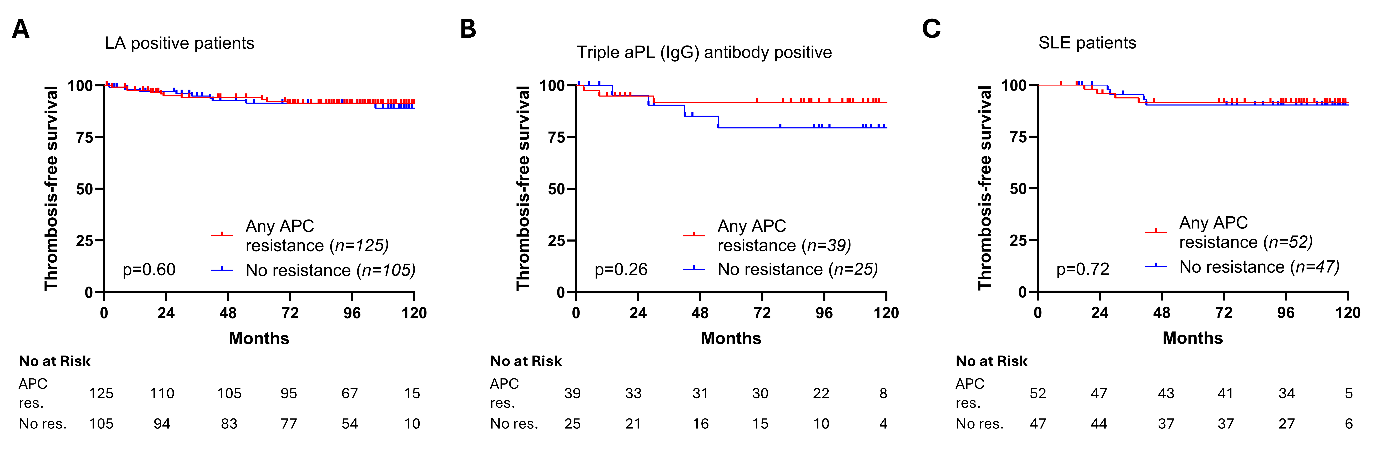


**Supplementary Figure S7. Activated protein C resistance does not predict future thrombosis in patients with high-risk antiphospholipid antibody (aPL) profiles or systemic lupus erythematosus (SLE).** Kaplan-Meier thrombosis-free survival analysis of prospectively followed patients within the antiphospholipid syndrome alliance for clinical trials and international networking (APS ACTION) registry with or without resistance to either recombinant human APC or Protac who were lupus anticoagulant (LA) positive (A), triple aPL (IgG subtype only) positive (B) or aPL-positive patients with SLE, which includes SLE-like disease (C). The dotted lines represent the 95% confidence intervals. No at risk – number at risk (those that are remaining without event or being censored)


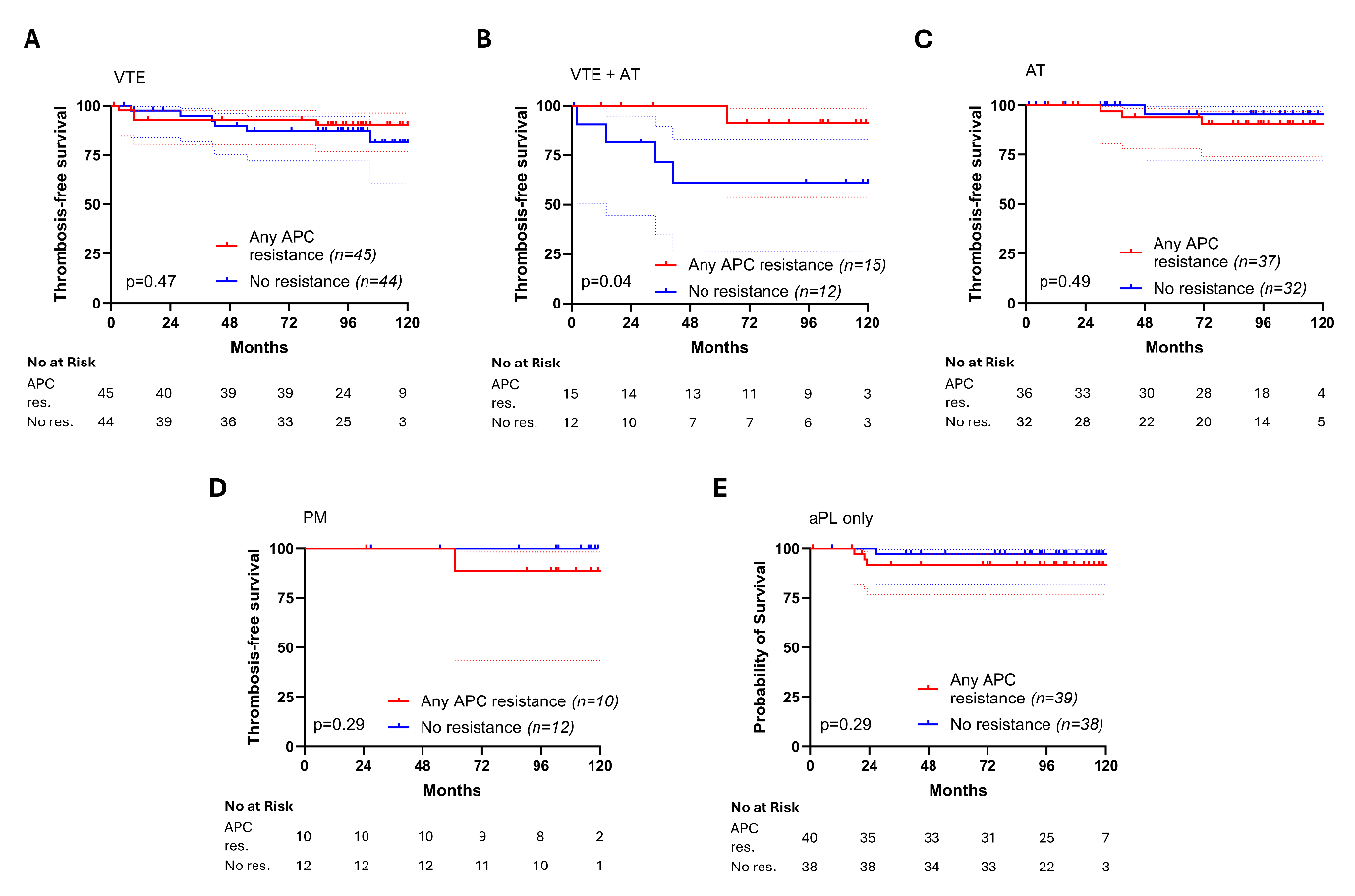


**Supplementary Figure S8. Activated protein C resistance does not predict future thrombosis in clinical subgroups of patients with aPL or APS.** Kaplan-Meier thrombosis-free survival analysis of prospectively followed patients within the antiphospholipid syndrome alliance for clinical trials and international networking (APS ACTION) registry with or without resistance to either recombinant human APC or Protac who had prior venous thromboembolism (VTE, A), VTE + arterial thromboembolism (VTE + AT, B), arterial thromboembolism (AT, C), pregnancy morbidity (PM, D), or were carriers of aPL only (aPL, E). The dotted lines represent the 95% confidence intervals. No at risk – number at risk (those that are remaining without event or being censored).
